# Surface Modification Using Assisting Electrodes in Wire Electrical Discharge Machining for Silicon Wafer Preparation

**DOI:** 10.3390/ma14061355

**Published:** 2021-03-11

**Authors:** Chunliang Kuo, Yupang Nien, Anchun Chiang, Atsushi Hirata

**Affiliations:** 1 Department of Mechanical Engineering, National Taiwan University of Science and Technology, #43, Sec. 4, Keelung Road, Taipei 106, Taiwan; m10903205@mail.ntust.edu.tw (Y.N.); m10503208@mail.ntust.edu.tw (A.C.); 2 School of Engineering, Tokyo Institute of Technology, 2-12-1 Ookayama, Meguro-ku, Tokyo 152-8550, Japan; hirata.a.aa@m.titech.ac.jp

**Keywords:** WEDM, assisting electrode, surface modification, material removal, surface topography, parametric optimization

## Abstract

This paper outlines notable advances in the wire electrical discharge machining of polycrystalline silicon workpieces for wafer preparation. Our use of assisting electrodes permits the transfer of aluminum particles to the machined surface of the polycrystalline silicon workpieces, to enhance conductivity and alter surface topography regardless of the silicon’s crystallographic structure and diamond-type lattice. This in-process surface modification technique was shown to promote material removal and simultaneously preserve the integrity of the machined surfaces with preferable surface textures. In the validation experiment, the 25 mm-thick assisting electrodes deposited a notable concentration of aluminium on the machined surface (~3.87 wt %), which greatly accelerated the rate of material removal (~9.42 mg/s) with minimal surface roughness (S_a_ ~5.49 μm) and moderate skewness (−0.23). The parameter combination used to obtain the optimal surface roughness (S_a_ 2.54 μm) was as follows: open voltage (80 V), electrical resistance (1.7 Ω), pulse-on time (30 μs), and electrode thickness (15 mm). In multiple objective optimization, the preferred parameter combination (open voltage = 80 V, resistance = 1.4 Ω, pulse-on time = 60 μs, and assisting electrode thickness = 25 mm) achieved the following appreciable results: surface modification of 3.26 ± 0.61 wt %, material removal rate of 7.08 ± 2.2 mg/min, and surface roughness of S_a_ = 4.3 ± 1.67 μm.

## 1. Introduction

The preparation of semiconductor wafers involves a series of manufacturing processes, including seeding, crystal growth, slicing into wafers, grinding, chemical-mechanical polishing, electrical-chemical etching, and coating [1,2,3,4,5]. Table 1 lists the comparisons of the methods in the processing of silicon wafers [6,7,8,9,10,11,12,13,14,15,16,17,18,19,20,21]. The extreme hardness (~10 GPa) and strength (170 GPa) of the monocrystalline substrate materials necessitates the use of wire-saw slicing [6,7,8,9,10,11] and diamond grit grinding [12,13,14,15,16]. Diamond-coated wire saws slice through the silicon ingot easily; however, the low fracture toughness (0.7 MPa·m^1/2^) often leads to collapse pits [6,7,8,9,11], cracking [7,8], and scratching grooves [6,7,8,9,10,11]. This has led to the development of alternative solutions aimed at reducing the contact force. Similarly, diamond-grit grinding delivers a consistent machined surface quality (R_a_ 0.07–3.35 μm) [12,13]. Nevertheless, cracking [12,14,15] and breakage [15] usually occur, particularly when separating the ground workpiece from the ingot. In contrast, wire electrical discharge machining (WEDM) has been shown to eliminate most contact forces, with acceptable results on the machined surface finish quality (R_a_ 2.31–6.3 μm) [17,18,19,20,21] and appreciable material removal rates (0.8–22.3 mm^3^/min) [17,18,19,21].

WEDM induces an electrical-thermal energy event, involving the transformation of electrical energy into joule heat, sufficient to melt a target region ahead of the wire electrode as it progresses through the electrically-conductive workpiece. The process of melting the silicon within this narrow discharge zone enables the removal of material with almost no wear on the electrode, regardless of the material’s hardness. When dealing with semi-conductive materials, the conversion of electrical-thermal energy in stochastic discharging actions can be only anticipated conditionally, which means that occasional under-discharging or over-discharging cannot be avoided. Huijun et al. [22] compared the effects of thermal power density in the WEDM processing of monocrystalline silicon within the context of the Joule-Lenz law. They estimated that the lowest energy consumption required to produce a cavity of ϕ: 20 μm within the discharge channel was 2.1 × 1012 W/cm^3^ under a drawn current of 1 A. The estimated energy density in the WEDM processing of monocrystalline silicon could perhaps be 10 orders of magnitude higher than the 108–109 W/cm^3^ required for common metallic materials. If the power consumptions were limited, then the extremely short discharging duration (~30 μs) of the pulse-on phase would be insufficient for the efficient removal of material. Dongre et al. [23] reported that a high servo voltage (~87 V) could significantly slow down the cutting speed by delaying the discharging actions, and thereby reducing the rate of material removal. To overcome metallurgical issues, Tian et al. [24] proposed cutting monocrystalline silicon along specific crystallographic planes in order to decrease the ohmic contact and improve machining efficiency. Using 261 mJ of electrical-thermal energy, they achieved cutting efficiency of 20,000 mm^2^/min, albeit at the cost of high surface roughness (R_a_ 5.5 μm). Ge et al. [25] investigated the WEDM processing of p-type silicon on the (100), (110), and (111) planes using a pulse-on time of 30–40 μs. This was shown to deteriorate the material to a depth of ~118 μm due to the long pulse duration, which provided sufficient time for transferring heat along the close-packed planes. As a result, unbalanced thermal stress across these specific planes having dissimilar thermal properties can lead to cracking along machined surfaces.

Surface modification to enhance the conductivity of discharging surfaces also makes it easier to estimate the electrical-thermal energy involved in discharging actions via theoretical analysis or empirical measurement. Reynaerts et al. [26] proposed superficial surface modifications involving the fabrication of temporary conductive layers using nickel or aluminium, with the aim of altering the electrical properties in order to perform discharging actions with minimal degradation to the machined surface. Other surface modification methods, such as doping, sputtering, and coating with extrinsic electro-conductive materials, can also be used to improve the electrical conductivity of semiconducting materials. Peng and Liao [27] studied the influence of doping p-type silicon ingots on WEDM slicing rates and surface roughness. Their highest slicing rate of 100.4 mm^2^/min was achieved using a pulse-on time of 0.6 μs coupled with a pulse-off time of 1.8 s and a drawn current of 360 A. Reducing the pulse-on time to 0.05 μs reduced the surface roughness to R_a_ 3.8 μm. Lee et al. [28] investigated the sputter deposition of aluminium on the surface of pure semiconducting germanium and the effect that this had on the WEDM slicing rate and subsurface damage. They found that an aluminium coating of 1 μm on the top and bottom surfaces increased the slicing rate to 7.7 mm^2^/min, which was 27 times faster than that achieved using uncoated germanium ingots. The metal coating on the outside surface enhanced electrical conductivity, which greatly improved the electrical discharging performance. Saleh et al. [13] investigated the performance of micro-EDM/WEDM machining when applied to a silicon workpiece coated with gold to a thickness of ~320 nm. Increasing the energy density to 360 nJ by applying an open voltage of 85–105 V and a 0.1–10 nF capacitor increased the material removal rate to 1.5 × 10^−3^ mm^3^/min, which is 23% higher than that of an uncoated silicon workpiece. Takino et al. [29] observed the surface damage incurred when using WEDM to cut workpieces of single-crystal silicon coated with electro-conductive resin masks (acryl resin mixed with silver powder) on the top and bottom surfaces. In the creation of bonding elements via WEDM, they recommended using oil-based dielectrics, such as silicon-silver composite, instead of water dielectrics which could migrate from the electro-conductive resin into the matrix. In contrast, Kuo et al. [30] investigated the use of assisting electrodes to coat an SKD11 workpiece with aluminium while simultaneously performing continuous WEDM cutting actions.

In this work, surface modification on p-type polycrystalline workpieces was evaluated through the material removal process of WEDM by sandwiching assisting electrodes (A6061-T6). Migrations of the elements from the assisting electrodes for synchronised surface modification in the material removal process were used to enhance material removal, reduce the machined surface roughness and alter the machined surface textures. The modified skewness and kurtosis of machined surface patterns were identified qualitatively and quantitatively for the successive chemical-mechanical planarization. Thermal damages on the machined surfaces such as chipping-off, craters, and cracks were discussed and reported. Parametric optimizations and statistical analysis in ANOVA and main effect plots were used to derive preferable parameter sets for each objective function. Finally, the optimization of the developed multi-objective function was mathematically demonstrated, and the obtained parameter sets were validated in experiments.

## 2. Experiment Procedures and Data Collection

### 2.1. Machining Strategy and Surface Modification

**Figure 1:**
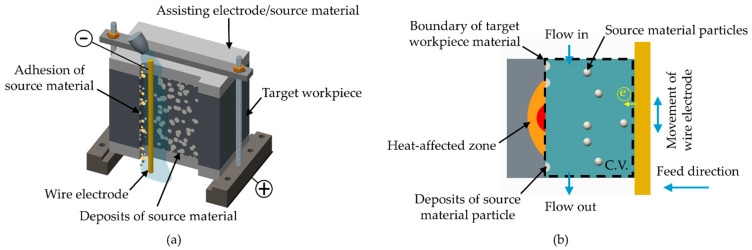
Schematic illustrations showing synchronized surface modification set-up: (**a**) use of assisting electrodes; (**b**) processes occurring in control volume during wire electrical discharge machining (WEDM).

Figure 1a illustrates the proposed stacking of materials for WEDM. The assisting electrodes (i.e., the upper and lower layers) supply the material to be transferred onto the target workpiece (middle layer). As the wire electrode moves reciprocally along the Z axis and advances into the target workpiece, discharging actions produce the high temperatures required to melt the target workpiece as well as the source material, resulting in the synchronized surface modification across the entire discharge area. As shown in Figure 1b, surface modification within the control volume depends on the flow of source material into the discharge area in the form of particles or debris; which is re-melted and then deposited across the surface of the workpiece. A discharging temperature that is too low would produce superficial deposits. A discharging temperature that is too high would allow the penetration of foreign materials into the subsurface through chemical reactions between the workpiece material and dielectric, thereby altering the composition of the workpiece.

**Figure 2:**
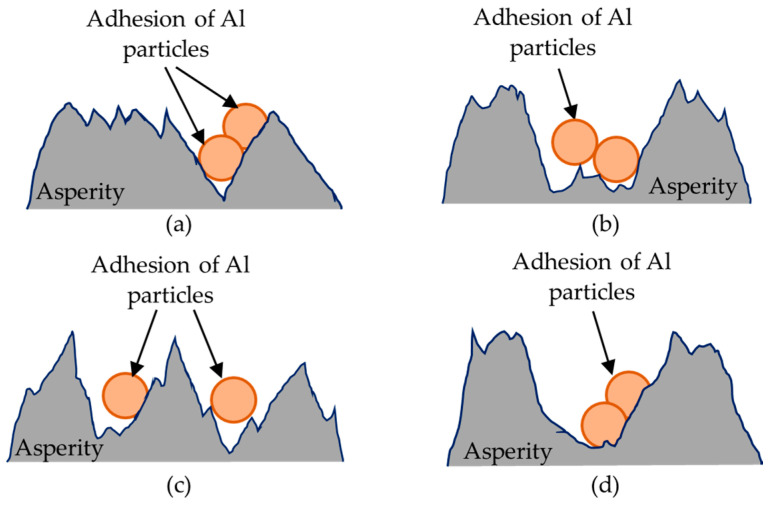
Schematic illustrations of expected surface topographies following the adhesion of particles on machined surface during WEDM: (**a**) negative skewness; (**b**) positive skewness; (**c**) high kurtosis; (**d**) low kurtosis.

Figure 2 depicts the expected cases of synchronized surface modification under the effects of cyclic discharging actions, involving the removal of source material and its subsequent deposition in the form of particles on the surface of the workpiece. Source material deposition is influenced by the current density and the availability of the source material. The regions in which the source material particles adhere can be classified using the terms positive skewness (Figure 2a), negative skewness (Figure 2b), high kurtosis (Figure 2c) and low kurtosis (Figure 2d). With continuous WEDM surface modification, the presence of the particles enhances the discharging actions by temporarily increasing the conductivity of the Si ingot; which increases the material removal rate and helps to preserve the machined surface. Parametric optimization of open voltage and pulse-on/off time to reduce the rate of flushing enables the texturing of machined surfaces to promote particle retention and facilitate subsequent manufacturing processes. Note that the high skewness topography provides pockets capable of retaining the chemical agents used in chemical-mechanical planarization (CMP) to enhance the polishing performance.

### 2.2. Stacked Materials, Wire Electrodes, and Machine Set-Up

**Figure 3:**
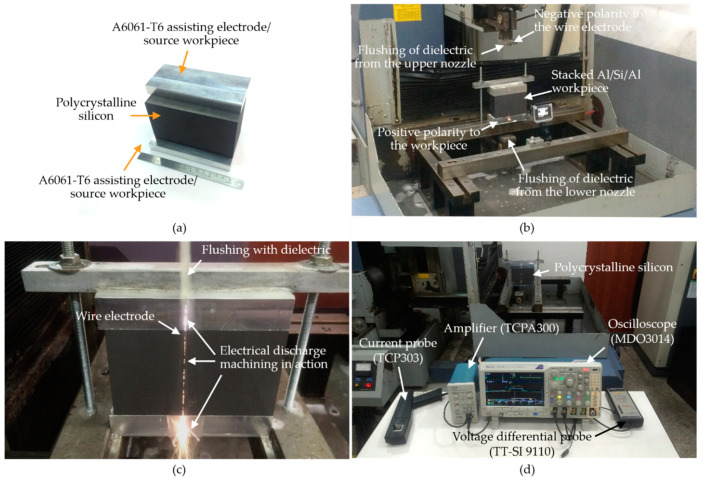
Set-ups for: (**a**) silicon workpiece sandwiched with assisting electrodes; (**b**) material removal and surface modification process; (**c**) synchronized surface modifications during wire electrical discharge machining; (**d**) wave form data acquisition in WEDM.

Figure 3a presents the target workpiece of polycrystalline silicon (156 × 100 × 156 mm^3^) sandwiched between assisting electrodes of aluminum alloy (160 × 100 × 0/15/25 mm^3^) during a discharging action. Table 2 details the physical and engineering properties of the target workpiece, the wire electrode, and the assisting electrodes used in the experiments. Figure 3b illustrates a test performed using a modified WEDM system with maximum current of ~50 A and an open voltage of 100 V. When using a controlled pulse-on time of 30–60 μs and pulse-off time of 150–300 μs, the system achieved a feed rate of ~50 μm/s. The dielectric emulsion was mixed with distilled water at a volume fraction of 40%, which was supplied at a flow rate of ~8 L/min to facilitate chip evacuation and heat dissipation. Figure 3c shows the electrical discharge machining in action using the molybdenum alloy wire electrode with a diameter of 0.20 mm and a constant load of 10 N in tension to retain stability. Figure 3d illustrates the set-up used for the measurement of the electrical signals (voltage and amperage wave forms) throughout the test intervals. Specimens cut from the stacked workpiece had cubic dimensions of 5 × 1 × 156 mm^3^. The specimens were carefully examined to reveal indications of surface deposits, alloying with source materials, the removal of source and target materials, and the topography of the machined surfaces.

**Table 2:** Properties of target workpiece, wire electrode, and assisting electrode material [31,32,33].

	Polycrystalline Silicon [31]	Mo [32]	A6061-T6 [33]
Density (g/cm^3^)	2.33	10.3	2.7
Hardness	7.0 Mohs	250 HV	95 HB_500_
Specific heat capacity (J/g·K)	0.71	0.25	0.896
Thermal conductivity (cal/cm × s × °C)	0.30	0.33	0.4
Electrical resistivity (μΩ·m)	10	0.19	0.04
Melting point (°C)	1412	2610	582–652

### 2.3. Designed Discharging Circuit

**Figure 4:**
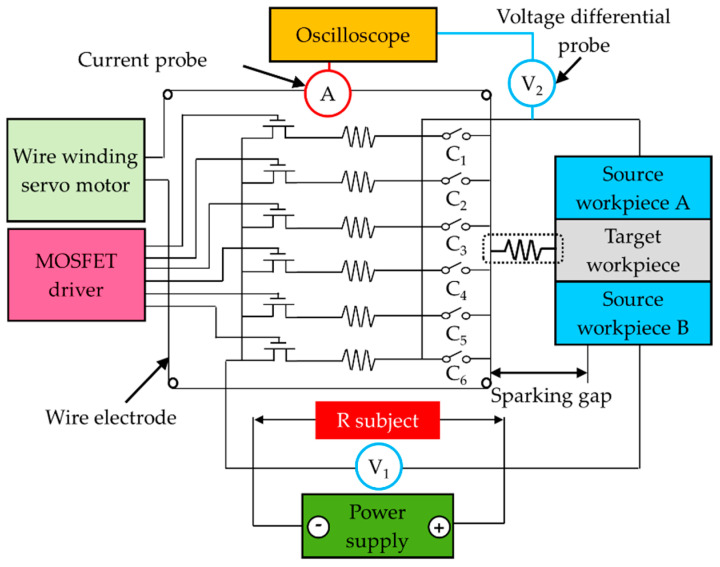
Designed integrated circuit modified to use MOSFET for high-power bipolar switching driver.

When the surface modification is successfully implemented, the discharging action in the semiconductor of polycrystalline silicon becomes similar to that of a normal metallic workpiece material. By means of sandwiching the source materials in a tandem arrangement with the target material, surface modification can be obtained by transferring the elements from the source materials to the successive target material in wire electrical discharge machining. Hence, the surface condition can be adjusted and altered [34,35]. In the baseline test, the recorded electrical resistance (Ohm) in the measured 50-mm span distance on the machined surface indicated a great reduction from ~70 kΩ∙m to 661 μΩ∙m due to the successful surface alloying. Figure 4 details the design of the discharging circuit with utilization of MOSFET drivers (Fairchild IRFP250, Sunnyvale, CA, USA) for high-power switching with bipolarities. The operating conditions of the proposed circuit include drawn electrical currents of ~25 A, an open voltage of 100 V, and drain-to-source resistance (rDS) of 0.085 Ω. The proposed circuit enables modification of the discharging profile by adjusting the voltage load and pulse-on time. By adjusting electrical resistance (R subject), the predetermined gap voltage (25 V) could ensure the drawn current at a consistent level of energy in breakdown avalanche mode with control over the sparking gap within a distance of up to 400 μm. The breakdown voltage and current values were recorded using an oscilloscope for the analysis of discharge waveform profiles.

### 2.4. Design of the Experiments

The transformation of electro energy into the thermal energy required for material removal is dominated by open voltage (V), electrical resistance (Ω), pulse-on time (t_on/off_) and the assisting electrodes’ (source workpiece) thickness. Source workpiece thickness, electrical resistance, open voltage, and overall resistance in the circuit (R subject) were control variables; whereas the polarity of the workpiece (+), the height of the target workpiece (5 mm), gap voltage (25 V), and feed rate (1.8 μm/s) were constants. Table 3 lists the four control variables (3 × 2 × 2 × 2) in the experiment, which resulted in 24 tests in the full factorial design. The range of electrical resistance values in this study resulted in a theoretical current density ranging from 35.09 to 50.51 A, over a projected area of 28.08 mm^2^ normal to the wire electrode.

### 2.5. Data Collection

As shown in Figure 3d and Figure 4, the open voltage (V_1_) and drawn electrical current (A) during the WEDM processing were recorded with a voltage differential probe (TESTEC TT-SI9110, FFM, Frankfurt, Germany) and a current probe (Tektronix TCP 303, Beaverton, OR, USA) in order. The voltage signal and drawn current were detected for analysis using an oscilloscope (Tektronix MDO3014, Beaverton, OR, USA) under a high-sampling rate (~3 MHz). This made it possible to obtain: precise measurements of voltages in the discharging states, the onset of the drawn current, and delays in turning the current on/off. The weight of the removed material during cutting was averaged from three repeated measurements. The surface roughness and the deposits of foreign particles were measured using a laser interferometer (Keyence one-shot 3D VR-3100, Osaka, Japan) with a scanning electron microscope (JEOL JCM-6000, Tokyo, Japan). Modifications to the machined sub-surface (in terms of alloying and element migration) were examined using an energy-dispersive X-ray spectroscope (JEOL JCM-6000, Tokyo, Japan).

## 3. Results and Discussion

### 3.1. Analysis of Discharge Voltage and Current Waveforms

**Figure 5:**
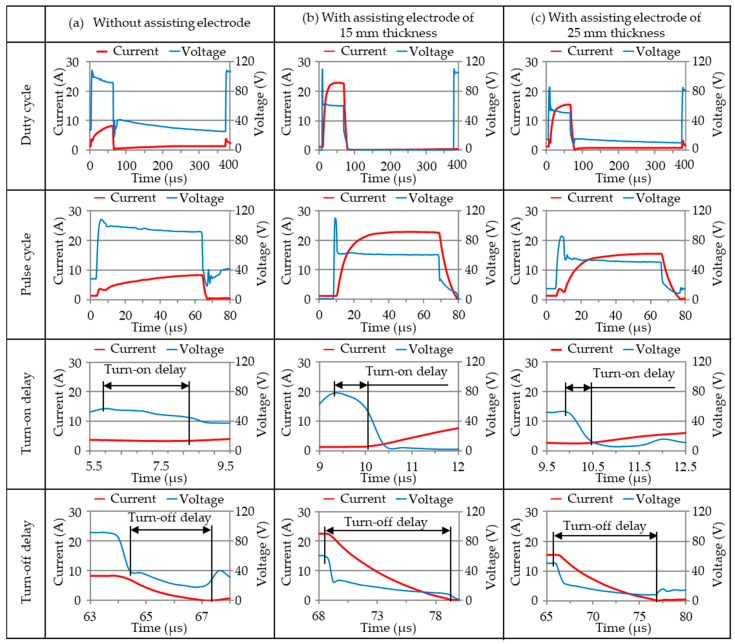
Measurements of discharge waveforms in WEDM of silicon workpieces using assisting electrodes of various thickness: (**a**) none; (**b**) 15 mm; (**c**) 25 mm, under the operating condition of 100 V, 1.7 Ω, and 60-μs pulse-on time.

Figure 5 presents the waveforms measured during discharge at an open voltage of 100 V, electrical resistance 1.7 Ω and pulse-on time of 60 μs using assisting electrodes with a thickness of 15 mm (Test 11) or 25 mm (Test 19) sandwiching polycrystalline silicon workpieces. Note that these values are compared with those in a typical operation without an assisting electrode (Test 20), which are usually influenced by the silicon’s crystallographic structure and diamond-type lattice. As shown in Figure 5a, a turn-on delay of ~3.4 μs (5.70–9.1 μs) at the onset of the discharging actions pushed out the current drawn in the pulse-on time interval; whereas a turn-off delay of ~3.2 μs (67.3–64.1 μs) continued nearly to the cessation of the test. This delayed response can be attributed to a discontinuity introduced by the breakdown of the dielectric insulation. Although the conductivity of the polycrystalline silicon workpiece was insufficient to guarantee a constant value in the discharging transient, the energy output in breakdown avalanche mode tended to increase while drawing only a small amount of current (~8.8 A). Figure 5b,c respectively present the relatively high currents (22.6 and 15.2 A) drawn when using the assisting electrodes. The corresponding turn-on delays were shortened (1.4 and 0.4 μs) and the turn-off delays were greatly increased (10.9 and 9.8 μs). The decrease in turn-on delay can be attributed to the low resistivity of the assisting electrodes (~0.04 μΩ·m), which increased the current drawn. Melting of the aluminum alloy electrodes during discharge could easily alter the surface of the polycrystalline workpiece via coating or deposition. The modified surface would tend to increase conductivity in the discharging region of the polycrystalline silicon workpiece, thereby promoting discharging actions via physical and/or chemical interactions. Increasing the size of the discharging area would increase the drawn current; however, it could also hinder the recovery of the insulation in the dielectric adjacent to the discharging zone, which could delay the initiation of the turn-off cycles, thereby necessitating a longer pulse-off cycle.

**Figure 6:**
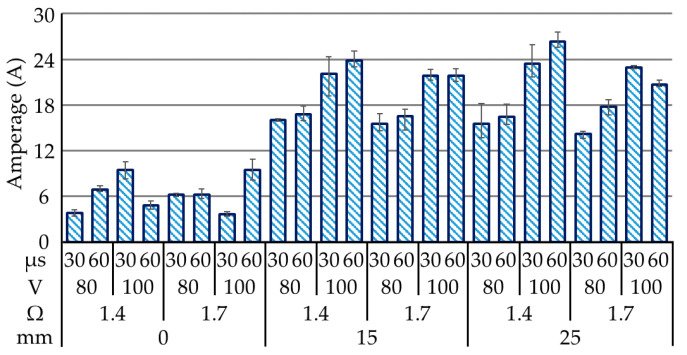
Measurements of discharge current in all 24 tests.

Figure 6 presents measurements of the average peak current values in each of the tests. We can see that without using electrodes, the drawn current was relatively low (3.63~9.41 A) since silicon’s crystallographic structure and diamond-type lattice would be selectively conductive on the preferable planes. The use of assisting electrodes dramatically increased the drawn current as follows: 15-mm electrode (15.53~23.80 A; +152%) and 25-mm electrode (14.17~26.35 A; +180%), regardless of the silicon’s lattice structure and the preferable orientation of the electrical conductivity. Without the assisting electrodes, none of the operating parameters had a noticeable effect on the amount of current drawn. This can be attributed to the conditionally conductive nature of the polycrystalline silicon (~80 MΩ), which is easily affected by the operating temperature and/or the content of the doped alloy (B, P, As, and Sb).

**Figure 7:**
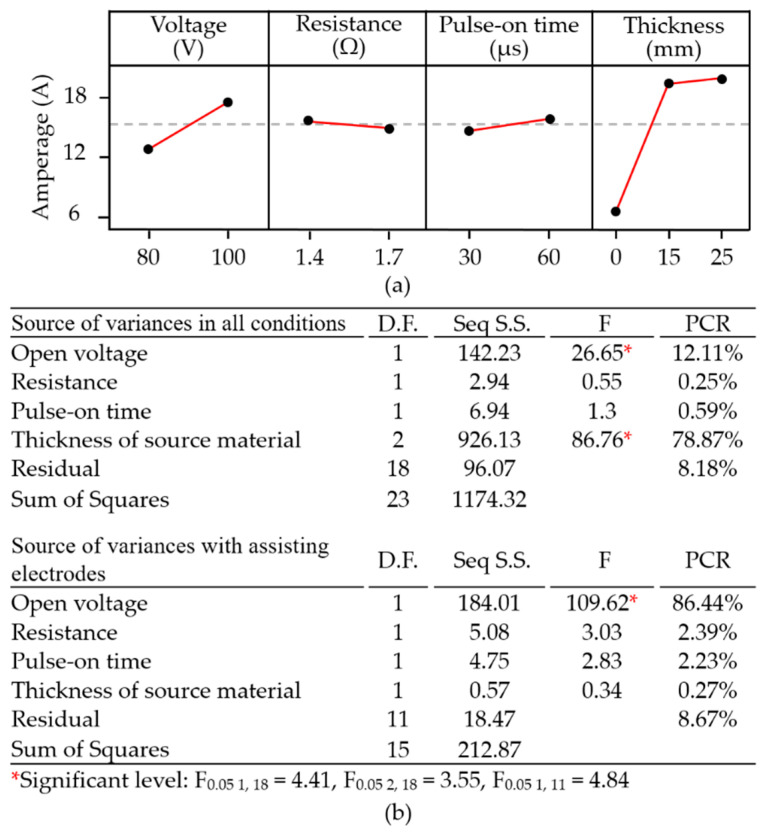
Statistical examination of main effect plots: (**a**) with operating parameter settings for assisting electrodes; (**b**) ANOVA results for discharging current.

The main effect plots in Figure 7a revealed that the thickness of the assisting electrodes was the dominant operating parameter; therefore, we obtained secondary main effect plots by blocking the null thickness of the assisting electrode. The combination of operating parameters associated with the amperage (~26.35 A) included a thick assisting electrode (25 mm), low electrical resistance (1.4 Ω), high open voltage (100 V) and long pulse-on time (60 μs), as validated in Test 23. The low electrical resistance and high open voltage increased the amount of current drawn. In contrast, the extended pulse-on time reduced the amount of current drawn due to the turn-on delay, which was limited by the MOSFET. Note that the aluminum electrode produced conductive particles, which were available for surface deposition in the sparking gap between the polycrystalline silicon workpiece and the wire electrode. The deposition of conductive material on the surface of the polycrystalline silicon increased its conductivity, leading to an increase in the amount of current drawn. The ANOVA (Minitab 17.1.0) results in Figure 7b indicate the strong influence of assisting electrode thickness on current draw, as indicated by the high percentage contribution ratio (PCR) of 78.87%. When comparing the effects of the drawn currents between the 15 and 25-mm assisting electrodes, the PCR (86.44%) of the open voltage greatly surpassed the other variables. The influence of electrical resistance and pulse-on time were overridden by those of source material thickness and open voltage, so long as the discharging energy was within the preferable range of 42.11–151.52 mJ/mm^3^. Extending the pulse-on time and reducing resistance could theoretically increase the current draw; however, these actions did not have a significant effect, perhaps due to the secondary deposition of aluminium and its effect on operating efficiency.

### 3.2. Surface Modification

**Figure 8:**
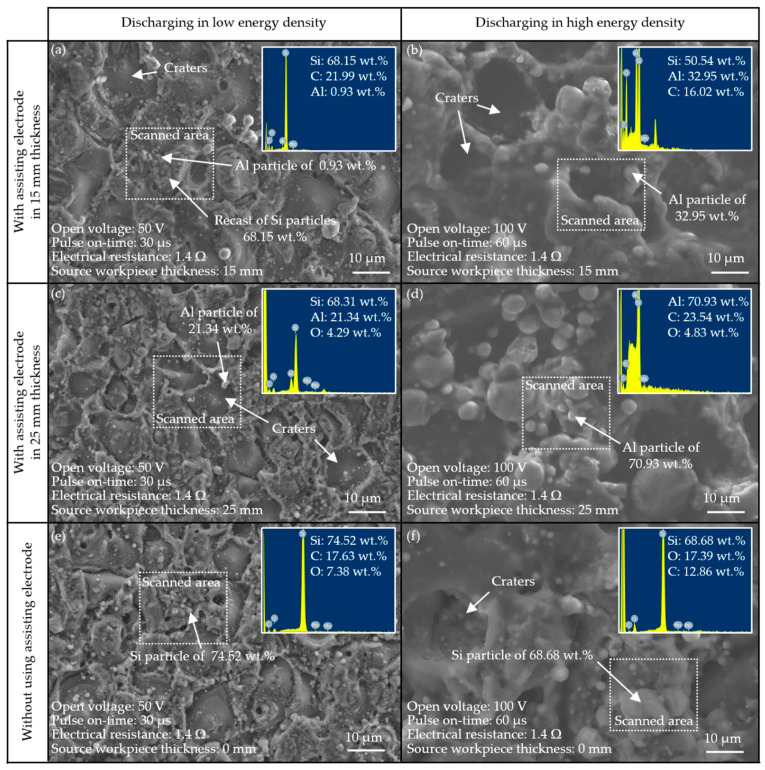
Surface morphology produced with: (**a**) low discharging energy density; (**b**) high discharging energy density coupling with 15-mm thickness electrodes; (**c**) low discharging energy density; (**d**) high discharging energy density coupling with 25-mm thickness electrodes; (**e**) low discharging energy density; (**f**) high discharging energy density without assisting electrode, and the corresponding EDX results.

Figure 8a,c present micrographs showing the results of surface modifications with the corresponding energy dispersive X-ray diffraction (EDX) diagrams. They reflected the weight percentage of the sporadically adhered aluminum particles on the machined surface of the polycrystalline silicon workpiece from baseline tests under the conditions of a low open voltage (50 V) and low pulse-on time (30 μs). In contrast, Figure 8b,d reflect the adhesion of the aluminum particles in a high conversion rate over the entire machined surface from the mainstream tests under the high open voltage (100 V) and high pulse-on duration (60 μs). In particular, high currents of ~27.3 A were drawn in Tests 17 and 23 and yielded the thermal energy of 151.52 mJ during discharging.

During the pulse-on interval, aluminum source material was transferred to the machined surface following conversion of electrical energy into joule heat via the discharging channel. The resulting interaction caused by the material removal and the deposition of molten aluminum particles on the target workpiece produced the machined surface topography with high surface roughness, and negative skewness. During the pulse-off time interval, the heated area was cooled down by the dielectric, resulting in the consolidation of the aluminum particles on the machined surface and possibly the high kurtosis topography. EDX analysis revealed the consolidation of molten aluminum particles and subsequent agglomeration into clusters. Figure 8e,f present the surface morphology showing only recasts of silicon oxide particles whilst aluminum assisting electrodes were not in use. In the macro-view of the machined surface on the silicon workpieces, none of cracking or chipping at the boundaries or edges were observed when using assisting electrodes. The surface modification of the adhesive aluminum particles could also possibly delay the heat dissipation on the machined surface and whereby ease the thermal effects from the stress variation and volumetric shrinkage at the discharging zone. As a result, defects on the machined surfaces were reduced after surface modification was demonstrated.

**Figure 9:**
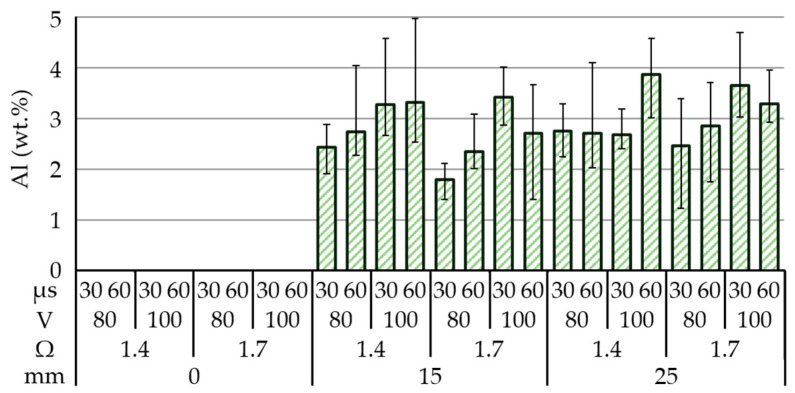
Surface modification of polycrystalline silicon as a function of source workpiece thickness.

Figure 9 indicates the amount of aluminum deposited on the machined surface: no assisting electrode (0%); 15 mm (1.80–3.32 wt %); and 25 mm (2.46–3.86%). In Test 17 (assisting electrode = 15 mm, open voltage = 100 V, electrical resistance = 1.4 Ω, pulse-on time = 60 μs) the distribution of aluminum across the 150-mm silicon workpiece was as follows: top (4.06 wt %), middle (3.12 wt %), and bottom (2.78 wt %). Increasing the thickness of the assisting electrode under the same operating parameters altered the distribution of the deposited aluminum from 2.47 to 3.87 wt %. Test 23 (assisting electrode = 25 mm, open voltage = 100 V, resistance = 1.4 Ω and pulse-on time = 60 μs) resulted in the highest concentration of aluminum (3.86 wt %). Interestingly, the deposition of aluminum did not increase proportionally with the thickness of the assisting electrodes, but rather decreased rapidly. This may be attributed to the fact that the drawn current did not increase proportionally with the thickness of the assisting electrodes. For example, the 25-mm electrode in Test 23 drew ~22.58 A, whereas the 15 mm electrode in Test 17 drew ~22.86 A. It is notable that increasing the thickness of the assisting electrodes decreased the average current density. Additionally, the discharging actions occurred stochastically over a larger area, such that they tended not to occur in the same spot or vicinity. Finally, the accumulation of particles in the discharging region reduced the current being drawn.

**Figure 10:**
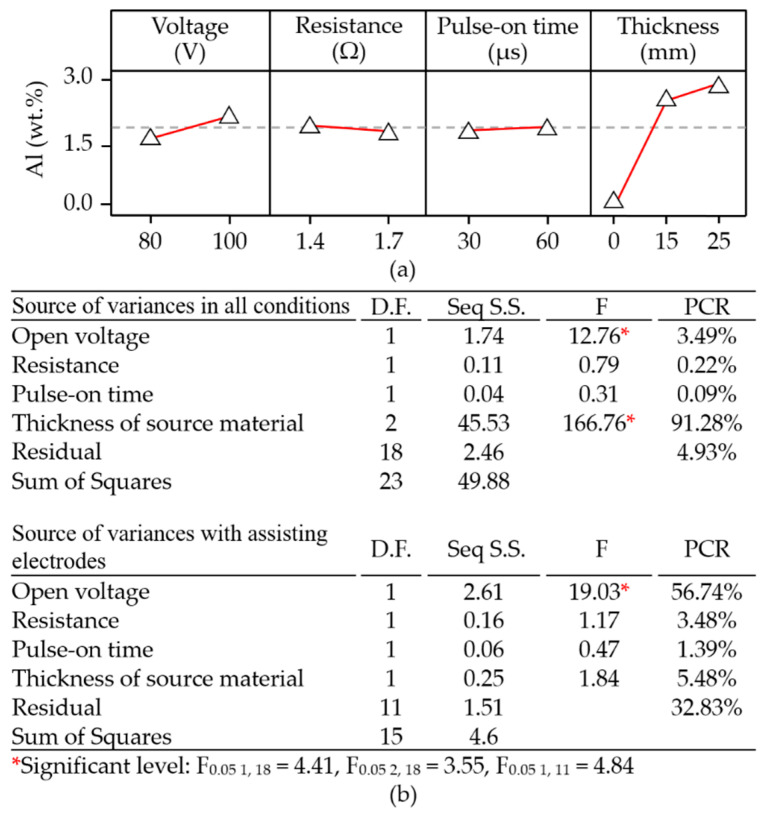
Statistical examination of main effect plots: (**a**) with operating parameter settings; (**b**) ANOVA results for deposition of aluminum on machined surface.

The main effect plot in Figure 10a revealed that a combination of low open voltage (80 V), high electrical resistance (1.7 Ω), long pulse-on time interval (60 μs), and thick assisting electrode (25 mm) was conducive to the deposition of aluminum. Under low electrical resistance conditions, the low voltage drew a high discharge current into a narrow sparking gap, thereby permitting the movement of aluminum particles onto the machined surface. This implies that a wider sparking gap (under high open voltage) may retard the movement of particles onto the machined surface. When using an extended pulse-on time interval, a thick assisting electrode would increase the current drawn and lead to the removal of more material for subsequent deposition during the electrical discharge interval.

### 3.3. Material Removal

**Figure 11:**
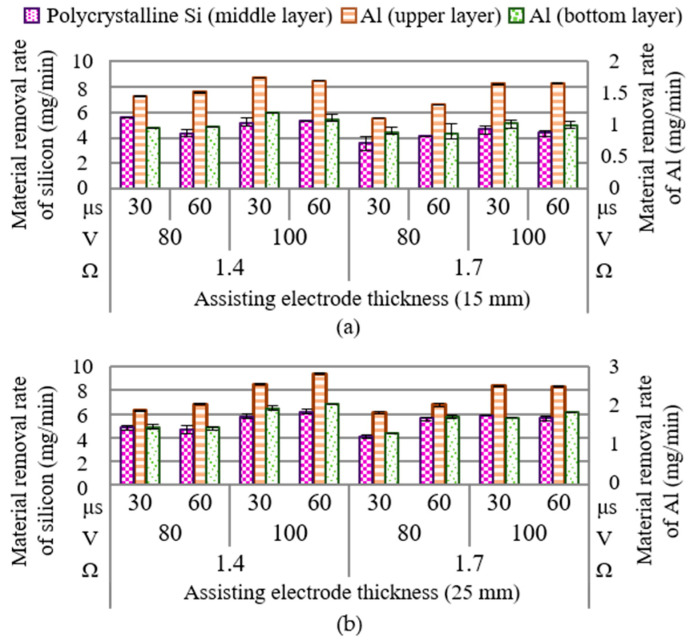
Material removal rate of polycrystalline silicon with assisting electrodes of: (**a**) 15 mm; (**b**) 25 mm.

Figure 11 illustrates the rate of material removal using assisting electrodes. As shown in Figure 11a, the rate of polycrystalline silicon removal ranged from 5.55–8.74 mg/min; whereas the rate of aluminum ranged from 0.71–1.11 mg/min in the upper electrode and 0.86–1.20 mg/min in the lower electrode. As shown in Figure 11b, increasing the thickness of the assisting electrode slightly increased the rate of material removal to 6.12–9.43 mg/min. The most rapid material removal (9.43 mg/min) was observed in Test 23 (assisting electrode = 25 mm, electrical resistance = 1.4 Ω, open voltage = 100 V, and pulse-on time of 60 μs), without any cracking or chipping off of the machined surface being observed.

In the WEDM processing of metallic materials, the governed mechanism underlying material removal is theoretically dominated by the current drawn and pulse-on time (discharge action), as these are the two main factors contributing to the energy density. However, a non-parametrical controlled factor of flushing could usually disturb the drawn current within the discharging gap, and thereby interfere with the theoretical anticipated factors. In particular, when the flushing actions are effectively undergone, the removed massive particles and debris are evacuated and will not be effectively adhered on the machined surface; leading to the reduction of the produced electrical conductivity on the machined surface, and the corresponding drawn current. In contrast, if the removed particles and debris are trapped on the machined surface, the electrical conductivity and drawn current will be conversely increased. Assisting electrodes are meant to enhance the conductivity of the polycrystalline silicon workpiece, in order to facilitate the movement of current for discharging actions, with a corresponding increase in discharging temperature when the pulse-on time and open voltage are parametrically controlled. The pulse-on time must exceed the turn-on delay interval to ensure the movement of sufficient current between the wire electrode and polycrystalline silicon workpiece. Nonetheless, if the pulse-on interval was too long, then the proportion of removed particles would be large, which would reduce the average current drawn, leading to an inefficient duty cycle and resulting in a low material removal rate. In contrast, if the set pulse-on interval was shorter than the turn-on delay time, the insufficient operating duration would not be efficient for drawing current to the peak values and would not be able to activate the discharging actions in effective ways. As a result, the material removal rate would not be successfully increased.

**Figure 12:**
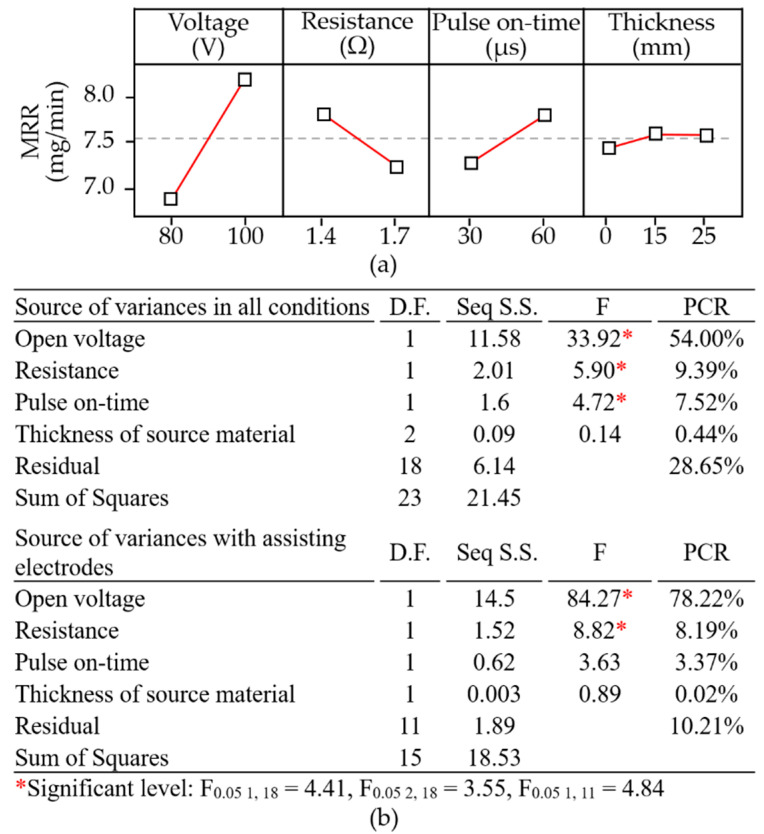
Statistical examination of main effect plots: (**a**) with operating parameter settings; (**b**) ANOVA results pertaining to material removal rate.

Figure 12a presents the parameter combinations conducive to material removal. Clearly, open voltage had the most pronounced effect on material removal (PCR = 54.00%); however, electrical resistance and pulse-on time also had significant effects. Note that the degree to which open voltage affected the material removal depended heavily on whether assisting electrodes were employed (PCR = 78.22%) or not employed (PCR = 54.00%). The ANOVA table in Figure 12b lists the statistical results pertaining to material removal. Clearly, when using assisting electrodes (15 or 25 mm), the rate of material removal increased with the open voltage. As discussed previously, the effect of the 15-mm electrodes on the surface modification was more pronounced than that of the 25-mm electrodes. Increasing the thickness of the electrodes to 25 mm led to a reduction in the current drawn under the effects of secondary discharging actions involving particles removed from the aluminum electrodes. Under these conditions, the amount of electrical power converted into joule heat was insufficient to complete the surface modification of the polycrystalline silicon workpiece, which compromised the discharging actions and the corresponding removal of material.

### 3.4. Surface Roughness

**Figure 13:**
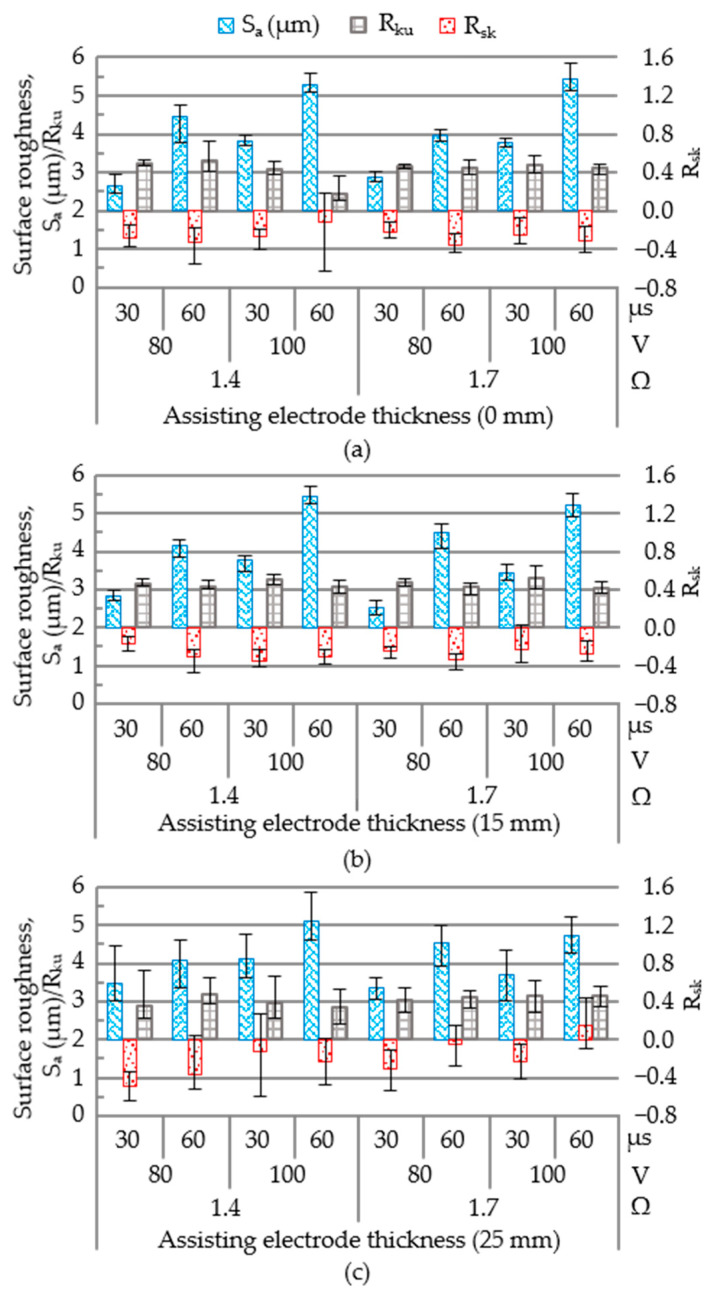
Surface roughness of polycrystalline silicon following WEDM processing: (**a**) without an assisting electrode; and with electrodes of (**b**) 15 mm; (**c**) 25 mm.

Figure 13 presents surface profiles obtained in all 24 tests, which covered a range of values, as follows: surface roughness (S_a_ 2.54–5.46 μm), skewness (R_sk_ −0.49 to 0.15) and kurtosis (R_ku_ 2.45–3.30). The lowest surface roughness (S_a_) was achieved using an open voltage of 80 V, electrical resistance of 1.7 Ω, and pulse-on time of 30 μs, regardless of the assisting electrodes’ parameters. Note however that surface quality depends on surface roughness as well as the deposition of molten aluminum particles in the form of aggregate and clusters, which can be described in terms of skewness (R_sk_) and kurtosis (R_ku_).

**Figure 14:**
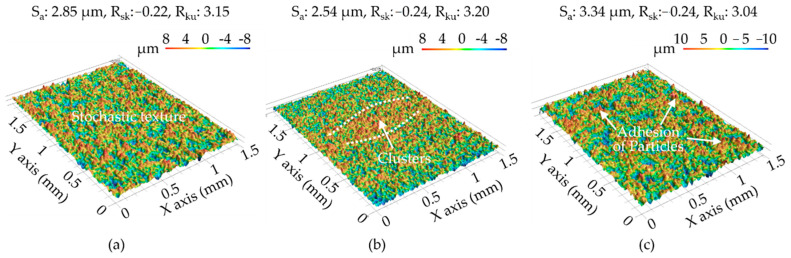
Three-dimensional (3D) photographic results following WEDM processing with open voltage of 80 V, electrode resistance of 1.7 Ω, and pulse-on time of 30 μs: (**a**) without assisting electrodes; and with assisting electrodes of (**b**) 15 mm; and (**c**) 25 mm.

**Figure 15:**
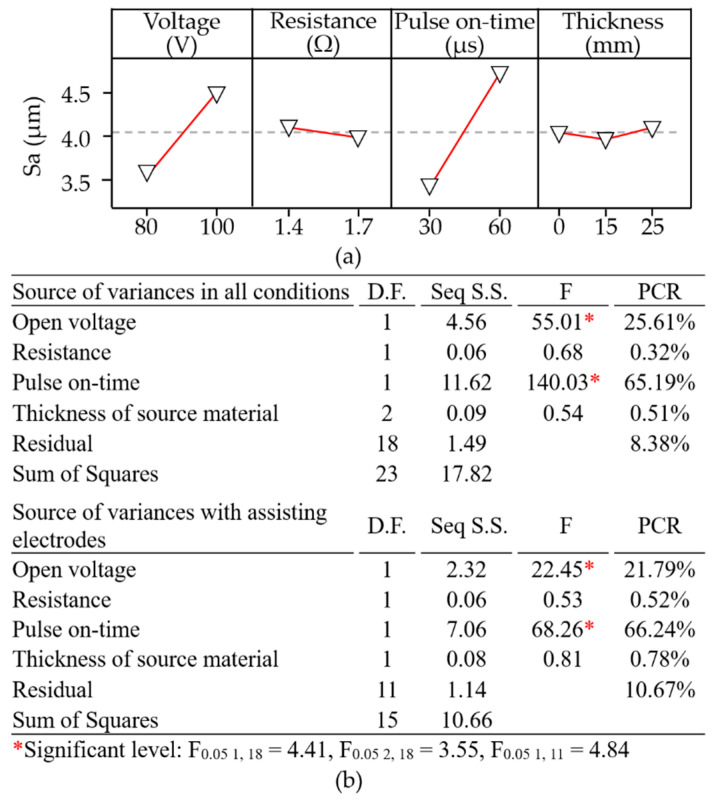
Statistical examination of: (**a**) main effect plots with operating parameter settings; (**b**) ANOVA results for surface roughness (S_a_).

As shown in Figure 14, samples prepared using a 15-mm electrode varied considerably in terms of surface elevation (R_ku_ = 2.45–3.30). The same samples presented distinct clusters of non-spherical aluminum particles (R_sk_ = −0.49 to 0.15). Figure 15a presents the main effect plots of surface roughness. The lowest surface roughness was achieved using an open voltage of 80 V, electrical resistance of 1.7 Ω, pulse-on time of 30 μs, and assisting electrode thickness of 15 mm. The ANOVA results in Figure 15b indicate that the open voltage had the most pronounced effect on the surface roughness (PCR = 25.61%), followed by pulse-on time (PCR = 65.19%). These results indicate that the deposition of foreign material (aluminum) did not have a pronounced impact on the average surface roughness. The skewness and kurtosis values presented consistent trends; however, they did not reveal a statistically significant influence on the average surface roughness. On the other hand, the preferable combination of operating parameters in WEDM of polycrystalline silicon workpieces could produce the adhesion of the aluminum particles on the machined surface of the polycrystalline silicon workpiece, and whereby increase the superficial electrical conductivity. Despite the wire electrical discharge machining of the polycrystalline silicon workpiece producing a degraded machined surface with recasts and craters, the preferable parametric control could yield surface topography characterised with a skewness of −0.49–0.15 and kurtosis of 2.45–3.30, which are conducive to trap aluminum particles on the machined surface of the polycrystalline silicon workpiece. As a result, the electrical conductivity on the machined surface was increased and hence, the drawn current would be increased and activate the effective joule heat transformation.

### 3.5. Multi-Objective Optimization

Following optimization of the parameter settings, we evaluated the criteria of the proposed WEDM. Table 4 lists the preferred parameter combinations for surface modification (Al wt %), material removal rate, and surface roughness, as well as the corresponding regression equations based on test results from the full factorial (3 × 2 × 2 × 2) experiment. Table 5 presents the superimposition of regression equations used to perform multi-objective optimization using an equal weighting factor of 33.33% for each objective function via dimensionless normalization. Four extreme values (A, B, C and D) were derived from the 50,127 process data points in the simulation space, based on the following operating parameters: open voltage (80–100 V; increments of 1 V), resistance (1.4–1.7 Ω; increments of 0.05 Ω), pulse-on time (30–60 μs; increments of 1 μs) and the thickness of the assisting electrode (0–25 mm; increments of 2.5 mm).

**Figure 16:**
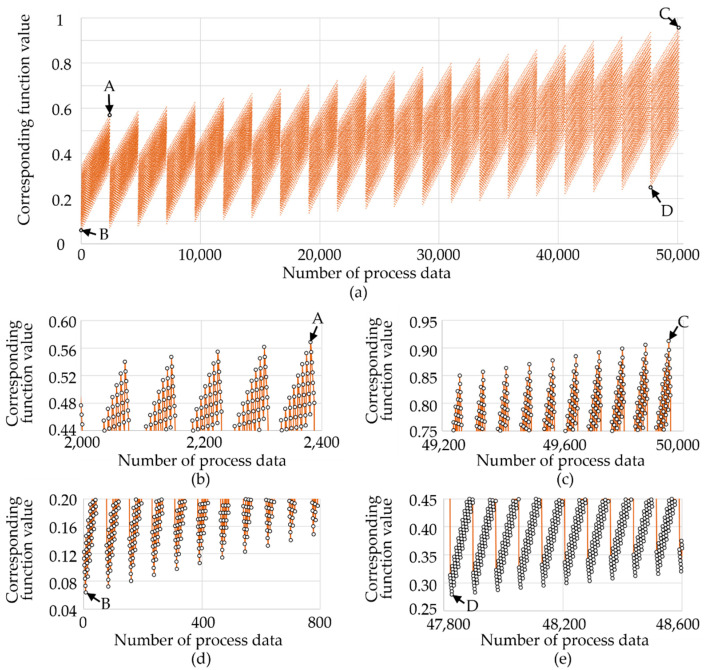
Scanning results: (**a**) performance of multi-objective optimization for each control interval and extreme values at (**b**) A point; (**c**) C point; (**d**) B point; (**e**) D point.

Figure 16 shows the scanning results in the controlled parameter intervals, indicating the four extreme values in the four corners. The interactions of the operating parameters produced saw-tooth profiles with envelopes at the top and the bottom of the plots; however, this did not significantly alter the slope or produce extreme outliers. Table 6 compares the optimized results obtained for single objectives and multiple objectives. The spans of the normalized deviation in the intervals were as follows: A (−24, 63), B (−98, 0), C (−12, 79), and D (−94, 19). The smallest span (87) was obtained using the parameter set in group A. Note that this parameter combination (open voltage = 80 V, resistance = 1.4 Ω, pulse-on time = 60 μs, and assisting electrode thickness = 25 mm) generated the least loss for any single objective. The experiment results were as follows: surface modification (3.26 ± 0.61 wt %), material removal rate (7.08 ± 2.2 mg/min), and surface roughness (S_a_ = 4.3 ± 1.67 μm).

## 4. Conclusions

This paper developed a novel approach using WEDM with assisting electrodes in a multilayer arrangement on the workpiece for slicing a silicon ingot. The research outcomes present novel technological and methodological innovations to facilitate material removal and preserve the finish of polycrystalline silicon workpieces. Statistical analysis of the experiment results has been used to identify the dominant factors affecting surface modification, material removal, and surface roughness, as well as the parameter combinations capable of optimizing these outcomes. Multi-objective optimization results based on mathematical analysis were then validated in the experiments. This work also advances our understanding of the mechanisms underlying electrical discharge-induced surface modification. The brief findings are as follows:

- The modified surface tended to increase conductivity in the discharging region of the polycrystalline silicon workpiece, thereby promoting discharging actions and drawing high currents (~25.35 A). The low conductivity of polycrystalline silicon (~10 μΩ·m) was overcome through application of conductive assisting electrodes (~0.04 μΩ·m) to reduce turn-on delays (~1.4 μs), albeit at the cost of increased turn-off delays (~10.9 μs).
- The deposition of aluminum did not increase proportionally with the thickness of the assisting electrodes, since the drawn current did not increase proportionally with their thickness. There was effective migration of aluminum to the machined surfaces when using assisting electrodes of 15 mm (1.80–3.32 wt %) and 25 mm (2.46–3.86%) over a sample area of 50 × 50 μm^2^.
- Assisting electrodes are meant to enhance the conductivity of the polycrystalline silicon workpiece when the pulse-on time sufficiently exceeds the recorded turn-on delay interval. When using assisting electrodes (15 or 25 mm), the parameter with the most pronounced effect on material removal was the open voltage (PCR = 78.22%). The optimal parameter combination in terms of material removal rate was as follows: high open voltage (100 V), high electrical resistance (1.4 Ω), long pulse-on time (60 μs), and thick assisting electrodes (25 mm).
- Machined surface quality in terms of skewness (R_sk_ = −0.49–0.15) and kurtosis (R_ku_ = 2.45–3.30) depended on the deposition of molten aluminum particles in the form of aggregate and clusters; however, the deposition of aluminum did not adversely affect the average surface roughness. The parameter combination used to obtain the optimal surface roughness (S_a_ 2.54 μs) was as follows: open voltage (80 V), electrical resistance (1.7 Ω), pulse-on time (30 μs), and electrode thickness (15 mm).
- In the multiple objective optimization, the preferred parameter combination (open voltage = 80 V, resistance = 1.4 Ω, pulse-on time = 60 μs, and assisting electrode thickness = 25 mm) achieved the following results: surface modification (3.26 ± 0.61 wt %), material removal rate (7.08 ± 2.2 mg/min), and surface roughness (S_a_ = 4.3 ± 1.67 μm).

## Figures and Tables

**Table 1:** Comparison of methods used in processing of silicon wafers [6,7,8,9,10,11,12,13,14,15,16,17,18,19,20,21].

Method	Surface Roughness (μm)	Material Remove Rate (mm^3^/min)	Challenges	Reference
Wire-saw slicing	R_a_ 0.14–4.23 [6,7,8,9,10,11]	28.7–94.4 [10,11]	Collapse pits [6,7,8,9,11] Fragmentation [7,8] Chipping-off [8] Scratching groove [6,7,8,9,10,11]	[6,7,8,9,10,11]
Diamond grinding	R_a_ 0.07–3.35 [12,13]	N/A	Grinding mark [12,14] Pitting [12,14] Crack [12,14,15] Chipping-off [16]	[12,13,14,15,16]
WEDM	R_a_ 2.31–6.3 [17,18,19,20]	0.8–22.3 [17,18,20,21]	Chipping-off [17] Crack [17,18,19] Crater [19,20,21] Bump [21]	[17,18,19,20,21]

**Table 3:** Test array.

Test No.	T_source workpiece_ (mm)	Electrical Resistance (Ω)	Open Voltage (V)	Pulse-on Time (μs)	Calculated Energy (mJ)
1	15	1.7	100	30	65.79
2	25	1.4	100	30	75.76
3	15	1.7	80	30	42.11
4	0	1.7	100	30	65.79
5	0	1.4	80	30	48.48
6	25	1.4	80	60	96.97
7	25	1.7	100	30	65.79
8	15	1.7	80	60	84.21
9	15	1.4	100	30	75.76
10	25	1.4	80	30	48.48
11	15	1.7	100	60	131.58
12	0	1.4	100	30	75.76
13	0	1.7	80	30	42.11
14	0	1.4	100	60	151.52
15	15	1.4	80	60	96.97
16	0	1.7	80	60	84.21
17	15	1.4	100	60	151.52
18	25	1.7	80	30	42.11
19	25	1.7	100	60	131.58
20	0	1.7	100	60	131.58
21	25	1.7	80	60	84.21
22	15	1.4	80	30	48.48
23	25	1.4	100	60	151.52
24	0	1.4	80	60	33.74

T_source workpiece_: Source workpiece thickness.

**Table 4:** Optimal parameter combinations for each individual objective.

Objective	Optimal Combination				Regression Equation
	x	y	z	k	
Surface modification (wt %)	100	1.4	60	25	f_surface modification_ (x, y, z, k) = −0.1 − 0.007·x − 1.13·y + 0.070·z − 0.022·k + 0.0199·x·y − 0.000383·x·z + 0.00152·x·k − 0.0239·y·z − 0.0020·y·k + 0.000334·z·k
Material removal rate (mg/min)	100	1.4	60	15	f_MRR_ (x, y, z, k) = 4.63 + 0.033·x − 0.49·y + 0.0841·z − 0.318·k − 0.0025·x·y − 0.000164·x·z + 0.003614·x·k − 0.0301·y·z + 0.0105·y·k − 0.000401·z·k
Surface roughness (μm)	80	1.7	30	15	f_Sa_ (x, y, z, k) = −6.91 + 0.1097·x + 2.61·y + 0.0139·z + 0.1364·k − 0.0393·x·y + 0.000186·x·z − 0.001016·x·k + 0.0158·y·z − 0.0077·y·k − 0.000652·z·k

x: open voltage (V); y: electrode resistance (Ω); z: pulse-on time (μs); k: electrode thickness (mm).

**Table 5:** Optimization equation and suggested parameter combinations.

Weighting Factor	Regression Equation	Parameters for Extreme Values				
		x	y	z	k	Group
Surface modification: 33.33% Material removal rate: 33.33% Surface roughness: 33.33%	F_multi-objective_ (x, y, z, k) = −0.7854 + 0.0448·x + 0.3267·y + 0.0305·z − 0.0527·k − 0.0072·x·y +0.0001·x·z + 0.0014·x·k − 0.0126·y·z + 0.0002·y·k − 0.0002·z·k	80	1.4	60	25	A
		80	1.7	30	0	B
		90	1.4	60	25	C
		90	1.65	30	0	D

x: open voltage (V); y: electrode resistance (Ω); z: pulse-on time (μs); k: electrode thickness (mm).

**Table 6:** Comparison of multi-optimization results with optimal combinations for single objectives.

Objective	Experiment Results for Single Objective	Modelling Value in Multi-Objective Equation	Deviation	Interval of Deviation
Group A				
Surface modification (wt %)	3.87	3.26	−16%	(−24, 63)
Material removal rate (mg/min)	9.28	7.08	−24%	
Surface roughness (μm)	2.63	4.3	63%	
Group B				
Surface modification (wt %)	3.87	0.09	−98%	(−98, 0)
Material removal rate (mg/min)	9.28	6.69	−28%	
Surface roughness (μm)	2.63	2.63	0%	
Group C				
Surface modification (wt %)	3.87	3.62	−6%	(−12, 79)
Material removal rate (mg/min)	9.28	8.18	−12%	
Surface roughness (μm)	2.63	4.71	79%	
Group D				
Surface modification (wt %)	3.87	0.24	−94%	(−94, 19)
Material removal rate (mg/min)	9.28	7.01	−24%	
Surface roughness (μm)	2.63	3.13	19%	

## Data Availability

Data sharing is not applicable to this article.
